# A translational-readiness framework for natural and engineered probiotics

**DOI:** 10.3389/fcimb.2026.1965075

**Published:** 2026-10-27

**Authors:** Nives Hribernik, Harsh Maan, Tatyana L. Povolotsky

**Affiliations:** 1 Institut für Chemie und Biochemie, Freie Universität Berlin, Berlin, Germany; 2 Department of Microbiology, NYU Langone Health Grossman School of Medicine, New York City, NY, United States

**Keywords:** clinical translation, engineered probiotics, live biotherapeutic products, microbial engraftment, next-generation probiotics, vaginal microbiome

## Abstract

The human microbiome has become a major source of therapeutic candidates, yet clinical translation has progressed far more slowly than mechanistic and preclinical discovery. In this review we ask: what determines whether a microbial therapeutic reaches the clinic? We compare traditional probiotics, next-generation probiotics and engineered live biotherapeutics using representative examples from metabolic disease, oncology and the vaginal microbiome. Together, these examples show that translational success depends less on biological sophistication than on the ability to achieve durable colonization, demonstrate meaningful clinical benefit and satisfy biocontainment and regulatory requirements. We explore the vaginal niche as a case study because it illustrates these challenges particularly well. Its relatively simple microbial community, local delivery and measurable ecological and clinical endpoints allow colonization, activity and recurrence to be assessed together. We integrate these observations into a translational-readiness framework that evaluates microbial therapeutics according to target specificity, colonization durability, biocontainment and safety, demonstrated benefit and regulatory precedent. This framework provides a practical approach for comparing emerging microbial therapeutic candidates and for identifying the factors most likely to accelerate their progression from promising biological discoveries to reliable clinical translation.

## 1 Introduction

The human gut harbors a dense microbial ecosystem comprising trillions of microorganisms whose collective genes far outnumber those of their host (Boulangé et al., 2016; Durack and Lynch, 2019). Far from passive commensals, these communities contribute to nutrient metabolism, immune development and colonization resistance. Disturbance of these communities, commonly termed dysbiosis, is associated with inflammatory bowel disease (IBD), type 2 diabetes mellitus (T2DM), colorectal cancer and other immune, metabolic and neuropsychiatric disorders (Boulangé et al., 2016; Durack and Lynch, 2019). Although many microbiome associations remain correlative, recent research has identified the microbiome as a source of therapeutic candidates (Sorbara and Pamer, 2022; Pribyl et al., 2025), renewing interest in probiotics, defined as live microorganisms that provide health benefits when administered in adequate amounts (Hill et al., 2014).

Traditional probiotics, mainly species formerly classified within the genus *Lactobacillus*, together with *Bifidobacterium* spp. and *Saccharomyces boulardii*, have been evaluated in thousands of studies over three decades. Reproducible benefit has been shown for only a limited number of indications, and effects remain highly dependent on strain identity, disease context and host characteristics. The American Gastroenterological Association (AGA) concluded in 2020 that evidence was insufficient to recommend probiotics for most gastrointestinal disorders; separately, its 2024 guidance on fecal microbiota-based therapies emphasized the low certainty of evidence for several indications (Su et al., 2020; Peery et al., 2024). These assessments concern broad clinical use rather than every indication, and specific applications retain support, as discussed in section 3.3. The limitation is also ecological rather than taxonomic, since the same genera behave differently when they are native to the target niche, a distinction developed in section 4.1. Empirical use of conventionally selected strains has therefore given way to a search for more defined and predictable microbial functions (Zmora et al., 2018; Pribyl et al., 2025).

To develop more targeted microbial therapeutics, two complementary strategies have emerged. Metagenomics, cultivation and multi-omic profiling have enabled identification of next-generation probiotics (NGPs), commensal-derived microorganisms selected for defined ecological or host-modulatory functions (ClinicalTrials.gov, 2026; Al-Fakhrany and Elekhnawy, 2024; Jadhav et al., 2026). In parallel, synthetic biology has transformed probiotic chassis organisms into programmable live biotherapeutics that sense disease-associated signals, deliver therapeutic payloads and incorporate mechanisms for controlled persistence or containment (Charbonneau et al., 2020; Duan et al., 2025; Sadhu et al., 2025).

Biological sophistication denotes the engineered or evolved capacity of a probiotic consortium to execute targeted molecular interactions, survive gastrointestinal transit and exert synergistic, strain-specific therapeutic outcomes within the host microbiome. It has nevertheless outpaced clinical translation, and few microbial therapeutics have achieved reproducible efficacy despite strong mechanistic rationale and extensive preclinical validation (Liu et al., 2024; Duan et al., 2025). Across these approaches, successful translation depends not only on microbial function, but also on whether that function can be delivered at the intended site, for the required duration and within a permissive ecological and host context, and whether it produces meaningful clinical benefit. In this review, we compare clinical successes and instructive failures across therapeutic strategies, use the vaginal niche as a case study and propose a framework for identifying barriers to clinical translation. This is a narrative synthesis rather than a systematic review. We searched PubMed and Web of Science for clinical-stage microbial therapeutics, prioritizing controlled trials, regulatory decisions and mechanistic studies published between 2020 and 2026, and examples were selected to illustrate distinct translational outcomes rather than to provide comprehensive coverage.

## 2 Background and recent developments

### 2.1 From probiotic function to therapeutic effect

Probiotic effects arise from multiple microbial functions and their interactions with ecological context and host responses rather than from any single beneficial trait (Dawson et al., 2025; Pribyl et al., 2025). Microbial metabolites, particularly short-chain fatty acids (SCFAs), are among the best-characterized mechanisms, with butyrate-producing organisms such as *Faecalibacterium prausnitzii* and *Roseburia* spp. influencing epithelial barrier function and immune regulation (Quévrain et al., 2016). Other mechanisms include pathogen exclusion, bacteriocin production and modulation of resident microbial communities (Sorbara and Pamer, 2022).

These functions do not necessarily translate into therapeutic efficacy. Human studies show that probiotic performance depends on strain-specific properties, baseline microbiome composition and the ability of an administered organism to remain active within, or establish in, a permissive ecological niche (Zmora et al., 2018).

### 2.2 Next-generation probiotics and early clinical translation

NGPs are commensal-derived microorganisms selected for defined ecological or host-modulatory functions identified through metagenomic and multi-omic analyses rather than through empirical use. Many are obligate anaerobes with specialized metabolic capabilities (ClinicalTrials.gov, 2026; Al-Fakhrany and Elekhnawy, 2024; Jadhav et al., 2026). *Akkermansia muciniphila* is an illustrative example of this class. Live bacteria, pasteurized preparations and the outer-membrane protein Amuc_1100 have shown beneficial effects on insulin sensitivity, epithelial barrier integrity and metabolic health in preclinical models and early clinical studies (Plovier et al., 2017; Al-Fakhrany and Elekhnawy, 2024; Jadhav et al., 2026). However, clinical responses may depend on baseline microbiome composition and the ability of the organism to establish within the recipient community. Trials now under way include *A. muciniphila* for glucose metabolism (NCT04797442) and as an anti-PD-1 adjuvant in cancer (NCT06865521) (ClinicalTrials.gov, 2026).

*F. prausnitzii* is among the most abundant commensals in the healthy colon at roughly 5%, is a major butyrate producer and is consistently depleted in IBD, colorectal cancer and metabolic syndrome (Effendi et al., 2022; Al-Fakhrany and Elekhnawy, 2024). Its anti-inflammatory activity, mediated by butyrate-dependent NF-κB suppression and secretion of a microbial anti-inflammatory molecule, has supported its development as a live biotherapeutic, including a recently initiated immuno-oncology trial (NCT06551272) (ClinicalTrials.gov, 2026). Other candidates, such as *Bacteroides fragilis*, *Christensenella minuta* and *Roseburia* spp., illustrate the growing emphasis on microorganisms selected for defined mechanistic functions rather than broad probiotic effects (Al-Fakhrany and Elekhnawy, 2024; Jadhav et al., 2026). *Clostridium butyricum*, a butyrate-producing spore former with decades of clinical use in Japan, has entered randomized oncology trials and is considered in section 3.3.

### 2.3 The synthetic biology toolbox for probiotic engineering

Engineered live biotherapeutics (eLBPs) represent a complementary strategy in which microbial chassis are equipped with synthetic circuits for programmable sensing, computation and therapeutic delivery (Charbonneau et al., 2020; Duan et al., 2025; Duhan et al., 2025; Sadhu et al., 2025). *Escherichia coli* Nissle 1917 (EcN) remains the most developed chassis, owing to its long clinical safety record, genetic tractability and compatibility with synthetic biology tools (Duan et al., 2025; Duhan et al., 2025).

Genome-editing tools have also expanded rapidly. CRISPR-Cas9 remains standard, while base and prime editing now allow precise single-nucleotide changes without double-strand breaks. Prime editing in EcN reached 50-67% efficiency in 2025, producing scarless edits with fewer off-target effects (Chen et al., 2025). This matters for regulatory review, because it reduces concerns related to residual selection markers or antibiotic resistance cassettes.

The most transformative advance has been the development of programmable gene circuits that couple environmental sensing to controlled therapeutic responses. Sense-and-respond systems allow a strain to detect signals, such as pathogen quorum-sensing molecules or inflammatory markers, and release a payload only when required. In a landmark study, EcN carrying a circuit that detects *Pseudomonas aeruginosa* autoinducers produced bacteriocins and lytic peptides and cleared the pathogen in murine gut infection, both therapeutically and prophylactically (Duan et al., 2025; Sadhu et al., 2025). Logic gates, toggle switches and kill-switch circuits add further layers of control, including biocontainment against uncontrolled environmental dissemination (Charbonneau et al., 2020; Sadhu et al., 2025).

Advances in biomaterial-based delivery have complemented strain engineering. Encapsulation platforms, hydrogel formulations and nanoparticle-based systems improve survival during gastrointestinal transit and enhance localized activity (Zhou et al., 2022; Duan et al., 2025). Bacterial extracellular vesicles have also emerged as promising cell-free platforms that may circumvent the challenges of long-term microbial colonization (Wei et al., 2025). Together these technologies greatly expand what microbial therapeutics can be designed to do, but they do not by themselves ensure that the engineered function is delivered with sufficient exposure to produce clinical benefit. **Table 1** summarizes the natural and engineered products discussed in the following sections, together with their target indication and current stage of development.

**Table 1:** Selected natural and engineered probiotic therapeutics in development or clinical use (2022-2026).

Strain/product	Strategy	Disease target	Development stage	Ref.
Rebyota™; Vowst™ (SER-109)	Microbiota-based consortia (rectal; oral spores)	Recurrent CDI	Approved (FDA, 2022 and 2023)	(Feuerstadt et al., 2022; Khanna et al., 2022)
EcN (SYNB1618, SYNB1934)	PAL + Phe transporter	PKU	Phase 3 (discontinued 2024)	(Synlogic Inc., 2024; Vockley et al., 2023)
*A. muciniphila* (live)	Natural NGP	T2DM, obesity	Phase 2 (baseline-dependent effect)	(Zhang Y. et al., 2025)
*B. animalis* MN-Gup + GOS	Synbiotic (NGP + prebiotic)	T2DM	Randomized trial (n=120)	(Zhang C. et al., 2025)
*A. muciniphila* (baseline)	Natural NGP, response biomarker	NSCLC, anti-PD-1	Observational cohort (n=338)	(Derosa et al., 2022)
CBM588 (*C. butyricum*)	Live biotherapeutic; checkpoint inhibitor adjunct	Metastatic renal cell carcinoma	Phase 1 randomized (two trials)	(Dizman et al., 2022; Ebrahimi et al., 2024)
*F. prausnitzii*	Natural NGP	IBD, immuno-oncology	Phase 1/2 (initiated)	(ClinicalTrials.gov, 2026; Effendi et al., 2022; Al-Fakhrany and Elekhnawy, 2024)
*B. fragilis* (SK08)	Natural NGP formulation	Ulcerative colitis	Clinical trial (China)	(Jadhav et al., 2026)
EcN (nanobody/GM-CSF)	Tumor-colonizing, immune payload	Colorectal neoplasia	Phase 1 (colonization); efficacy preclinical	(Gurbatri et al., 2024)
EcN (ECN-pE)	CAT + SOD; biofilm encapsulation	IBD (colitis)	Preclinical (murine)	(Zhou et al., 2022)
EcN (anti-*Pseudomonas*)	QS-responsive bacteriocin circuit	MDR *P. aeruginosa*	Preclinical (murine)	(Duan et al., 2025; Sadhu et al., 2025)
LACTIN-V (*L. crispatus* CTV-05)	Natural single-strain vaginal LBP	Recurrent BV; HIV-risk markers	Phase 2b (positive; partial colonization)	(Cohen et al., 2020; Hemmerling et al., 2025; Bloom et al., 2026)
Multi-strain *L. crispatus* (VIBRANT)	Rational multi-strain consortium	Recurrent BV	Phase 1 (completed)	(Potloane et al., 2026)
VS-01 vaginal synbiotic	Multi-strain + substrates, slow release	Vaginal microbiome conversion	Randomized trial (healthy volunteers)	(Ravel et al., 2025)
Engineered *L. jensenii*	Secretes cyanovirin-N (HIV entry inhibitor)	HIV prevention (vaginal)	Preclinical (macaque)	(Liu et al., 2006; Lagenaur et al., 2011; Brichacek et al., 2013)
PM-477; BNT331-EL	*Gardnerella*-targeted endolysin	Bacterial vaginosis	Preclinical (ex vivo)	(Landlinger et al., 2021; Tisakova et al., 2025)

BV, bacterial vaginosis; CAT, catalase; CDI, *Clostridioides difficile* infection; EcN, *E. coli* Nissle 1917; GOS, galactooligosaccharide; LBP, live biotherapeutic product; MDR, multidrug-resistant; NGP, next-generation probiotic; NSCLC, non-small-cell lung cancer; PAL, phenylalanine ammonia lyase; PKU, phenylketonuria; QS, quorum sensing; SOD, superoxide dismutase; T2DM, type 2 diabetes mellitus.

## 3 Translational readiness across strategies

### 3.1 A translational success case: recurrent CDI

The clearest positive benchmark for translational readiness comes from recurrent *Clostridioides difficile* infection (rCDI). Rebyota™, given rectally, and Vowst™, an oral spore-based product, are microbiome therapeutics that were FDA-approved in 2022 and 2023 respectively to prevent recurrence after antibacterial treatment of rCDI (Feuerstadt et al., 2022; Khanna et al., 2022). Their clinical development was facilitated because several favorable factors aligned: a defined pathogen, a clear post-antibiotic treatment window, a meaningful endpoint and substantial unmet need. Together, these factors provide measurable clinical benefit from microbiome restoration. Nevertheless, approval does not eliminate evidentiary uncertainty. The 2024 AGA guideline recommends fecal microbiota-based therapies only conditionally, and rates the certainty of evidence as low (Peery et al., 2024).

By contrast, engineered probiotics for inflammatory gastrointestinal disease are often more mechanistically sophisticated but much less clinically mature. In murine colitis, EcN engineered to overexpress catalase and superoxide dismutase and protected in a chitosan-alginate formulation reduced oxidative stress and supported epithelial repair (Zhou et al., 2022). Other strains deliver cytokines, nanobodies, antimicrobial peptides or metabolites (Charbonneau et al., 2020; Duan et al., 2025), but controlled human efficacy has not been shown. The most clinically advanced products are therefore not the most extensively engineered. Instead, they are those for which the therapeutic target, treatment window, delivery route and clinical endpoint align.

### 3.2 Lessons from clinical setbacks: NGPs and metabolic disease

Metabolic disease shows why demonstrating microbial activity is not enough. Synlogic engineered the EcN strains SYNB1618 and SYNB1934 to consume phenylalanine in the gut of patients with phenylketonuria. In the 14-day Synpheny-1 study, exposure to orally administered deuterium-labeled phenylalanine fell by 34% and 43% respectively, providing clear evidence of target engagement (Vockley et al., 2023). The pivotal Synpheny-3 trial was nevertheless discontinued in February 2024 after an internal review indicated it was unlikely to meet its primary endpoint, a decision unrelated to safety (Synlogic Inc., 2024). The program thus demonstrated biochemical activity in humans, but this did not translate into meaningful clinical benefit. Because detailed analyses were not disclosed, the precise reasons remain speculative.

A different limitation emerged in a 12-week randomized, double-blind Phase 2 trial of live *A. muciniphila* in 58 participants with overweight or obesity and T2DM. Body weight and HbA1c declined in both groups, with no significant between-group difference overall. In prespecified subgroup analyses, however, participants with low baseline *A. muciniphila* colonized efficiently and lost body weight, fat mass and HbA1c, whereas those with high baseline abundance colonized poorly and showed no comparable improvement. Fecal-transfer experiments in germ-free mice reproduced this pattern (Zhang Y. et al., 2025). These findings show that baseline microbiome composition can influence whether supplementation alters community structure or host phenotype.

This dependence on baseline ecology suggests that microbial performance may be improved by modifying the niche alongside the administered organism. In a randomized, double-blind trial of 120 patients with T2DM, *Bifidobacterium animalis* subsp. *lactis* MN-Gup combined with galactooligosaccharide lowered fasting blood glucose more than MN-Gup alone or placebo, though whether the advantage came from improved engraftment, greater local metabolic activity or another host-microbe interaction was not established (Zhang C. et al., 2025). Together these studies highlight two gaps: biochemical target engagement may not produce clinical benefit, and microbial performance may depend on the recipient’s baseline microbiome.

### 3.3 Promising but preliminary: oncology and emerging applications

In oncology the evidence is clinically relevant but still preliminary. Association between a microorganism’s abundance and treatment outcome does not establish that administering it will improve that outcome, and site-specific localization of an engineered bacterium does not demonstrate efficacy. In a prospective cohort of 338 patients with advanced non-small-cell lung cancer treated with immune-checkpoint inhibitors, baseline intestinal *A. muciniphila* was associated with higher objective response rates and longer overall survival in multivariable analyses, independently of PD-L1 expression and other established clinical factors (Derosa et al., 2022). Whether this association can be translated into therapeutic benefit is now being tested in early interventional studies of *A. muciniphila* supplementation (ClinicalTrials.gov, 2026).

Evidence is not confined to *A. muciniphila*, nor to next-generation organisms. A systematic review and meta-analysis of patients receiving immune checkpoint inhibitors found that probiotic supplementation, largely with conventional *Lactobacillus* and *Bifidobacterium* preparations, was associated with improved survival outcomes, although the authors emphasize that large, high-quality randomized trials remain necessary (Zhao et al., 2025). These findings underscore that conventional probiotics may have application-specific clinical value despite limited evidence across broader indications. *Clostridium butyricum* MIYAIRI 588 (CBM588) provides another example of the clinical potential of next-generation probiotics. In randomized trials, CBM588 improved progression-free survival when combined with immune checkpoint therapy in patients with metastatic renal cell carcinoma, despite no corresponding shift in the measured microbiological endpoints (Dizman et al., 2022; Ebrahimi et al., 2024).

A second strategy is to use engineered bacteria as local therapeutic delivery platforms. Orally administered EcN preferentially accumulated in colorectal neoplastic tissue in a double-blind pre-resection clinical trial, providing human evidence that the strain can reach the intended site. In the same report, an engineered strain releasing GM-CSF and nanobodies against PD-L1 and CTLA-4 reduced adenoma burden by approximately 50% in *Apc^Min/+^* mice (Gurbatri et al., 2024). These findings establish different levels of evidence: the human study demonstrated selective colonization, whereas therapeutic benefit remains preclinical. This distinction is notable because cancer outcomes are shaped by tumor genetics, immune status, prior treatment and disease burden (Derosa et al., 2022). Microbiome interventions may ultimately serve as response modifiers or local delivery platforms, but current evidence supports association and site-specific delivery more strongly than clinical antitumor benefit. Applications in the gut-brain axis, bone disease and xenobiotic metabolism remain exploratory (Ferrari et al., 2024; Emanowicz et al., 2025; Wei et al., 2025).

## 4 The vaginal niche as a test case for microbial therapeutics

### 4.1 Why the vaginal niche is different

The vaginal niche provides a more tractable setting for live biotherapeutics than most gut indications. Unlike the highly diverse and spatially complex intestinal microbiome, the healthy vaginal ecosystem is typically low in microbial diversity and often dominated by a limited number of *Lactobacillus* species, particularly *Lactobacillus crispatus*. Lactic acid produced by these communities maintains a low pH and restricts the growth of *Gardnerella* and other bacterial vaginosis-associated anaerobes (Ravel et al., 2011; O'Hanlon et al., 2013; Anahtar et al., 2015). The therapeutic target is therefore comparatively well defined: establishment and maintenance of a protective lactobacillus-dominant community. This also resolves an apparent tension with our assessment of conventional probiotics in section 1. The distinction is ecological rather than taxonomic. *L. crispatus* is native to the vaginal niche, where it can form a stable, protective community state. By contrast, conventional oral probiotic strains are introduced into the gut, where their persistence can depend strongly on the recipient’s microbiome. Thus, the same genus can have very different therapeutic potential depending on the organism and its ecological context (Kern et al., 2026).

The clinical rationale is also substantial. Bacterial vaginosis (BV) is often recurrent and is associated with increased susceptibility to sexually transmitted infections, including HIV, and with adverse pregnancy outcomes such as preterm birth. Although antibiotic treatment with metronidazole and clindamycin achieves short-term remission, it frequently fails to restore durable lactobacillus dominance, resulting in high recurrence rates (Leitich et al., 2003; Bradshaw et al., 2006; Atashili et al., 2008). Local administration delivers a product directly to the affected niche, while microbiome composition, vaginal pH and recurrence can be measured in parallel. Together, these features allow colonization, local activity and recurrence to be assessed in the same setting.

### 4.2 What the trials show and where they fall short

The strongest efficacy evidence comes from LACTIN-V, a vaginal live biotherapeutic containing *L. crispatus* CTV-05. In a Phase 2b trial of 228 women who had completed a course of vaginal metronidazole gel, BV recurred by week 12 in 30% of LACTIN-V recipients and 45% of placebo recipients, with benefit still evident at week 24 (Cohen et al., 2020). This is one of the clearest demonstrations of microbiome-based efficacy, linking a defined strain, targeted delivery, restoration of a protective community and a meaningful endpoint. However, benefit was achieved despite incomplete and variable long-term colonization, showing that clinical efficacy does not necessarily require durable engraftment in every recipient.

A separate Phase 2 study in young South African women examined mucosal and microbiological markers of HIV susceptibility. At week 4, *L. crispatus* dominance was seen in 41% of LACTIN-V recipients, and the post-metronidazole rise in activated endocervical CD4+ HIV target cells observed on placebo was absent. Colonization declined once dosing stopped, however, and the study was not designed to test HIV acquisition (Hemmerling et al., 2025). The findings therefore support restoration of a lactobacillus-dominated environment and favorable mucosal immune changes, but not prevention of HIV acquisition.

The central limitations are incomplete and variable engraftment. A 2026 multi-omic analysis of the Phase 2b trial found an *L. crispatus*-dominant microbiome at week 12 in 30% of treated participants against 9% on placebo, with colonization success associated with the pre-treatment microbiome and inflammatory state and with the bacterial load remaining after metronidazole (Bloom et al., 2026). This shows that the post-antibiotic niche is not equally suitable for colonization in all recipients. LACTIN-V can reduce recurrence, but it does not reliably establish long-term *L. crispatus* dominance.

### 4.3 Strategies to improve colonization and therapeutic function

Recent studies have aimed to improve the consistency and durability of *L. crispatus* colonization through rational strain selection, ecological support, optimized release and selective removal of competing organisms. The Phase 1 VIBRANT trial evaluated two multi-strain *L. crispatus* products after metronidazole in 91 participants with BV. Product strains were detected in 66% of active-group participants during the first five weeks, and 49% of initial colonizers still carried them at week 12 despite a short dosing course, usually as dominant members. Participants in whom product strains were detected also had a lower risk of recurrence (Potloane et al., 2026). However, because colonization was an observed outcome rather than experimentally assigned, the trial cannot establish that engraftment itself caused the lower recurrence.

Formulation offers another route. In a randomized study of 70 healthy premenopausal participants, the vaginal synbiotic VS-01 combined three *L. crispatus* strains with growth-supporting substrates and was given without antibiotic pretreatment. Among participants lacking an *L. crispatus*-dominant microbiome at baseline, 90% receiving the slow-release tablet converted to a dominant community by day 21, against 11% on placebo, and the tablet outperformed rapid-release vaginal and oral formulations of the same strains (Ravel et al., 2025). Although the study measured microbiome conversion rather than recurrence, it shows that release kinetics and ecological support can strongly influence microbial establishment.

Once stable colonization can be achieved, strains adapted to the vaginal niche may also be engineered to provide additional therapeutic functions. A natural vaginal isolate of *Lactobacillus jensenii* has been engineered to secrete a modified HIV-entry inhibitor, cyanovirin-N (Liu et al., 2006), and macaque studies showed vaginal colonization with continued transgene expression and no rise in mucosal inflammatory cytokines (Lagenaur et al., 2011; Brichacek et al., 2013). These studies provide proof of concept for combining ecological colonization with local therapeutic delivery, although human safety, stability and efficacy remain to be established.

A complementary approach is to make the niche more permissive before introducing beneficial strains by selectively removing competing organisms. *Gardnerella*-targeted endolysins clear dysbiotic biofilms alongside lactobacillus restoration. PM-477 eliminated *Gardnerella* and disrupted biofilms in 13 of 15 *ex vivo* samples while largely sparing the remaining microbiota (Landlinger et al., 2021). Its clinical successor BNT331-EL depleted viable *Gardnerella* by at least 94%, but also reduced *Lactobacillus iners* by an average of 92% (Tisakova et al., 2025). Selectivity across vaginal lactobacilli is therefore incomplete, although *L. crispatus* proliferated when already sufficiently abundant. Neither compound has yet shown clinical efficacy in humans.

The vaginal niche offers a clearer translational path than gut-directed applications, but success will require integrating microbial selection, ecological conditioning and precision delivery rather than simply administering beneficial strains. Key questions remain: how to achieve durable engraftment, identify recipients with a permissive post-treatment niche, maintain stable expression in engineered strains and establish safety for repeated use.

## 5 Grading translational readiness

### 5.1 Biocontainment and regulatory classification

Two additional factors, biocontainment and regulatory classification, receive less attention than efficacy but strongly influence whether a microbial therapeutic reaches patients.

Biocontainment is essential because engineered live biotherapeutics are introduced into complex microbial ecosystems and may persist unintentionally, undergo genetic changes or transfer engineered genetic material. Kill switches, auxotrophic dependencies and inducible expression systems provide important safeguards (Charbonneau et al., 2020; Sadhu et al., 2025), while scarless editing can avoid retaining antibiotic-resistance cassettes in the final construct (Chen et al., 2025). The long-term stability and evolutionary behavior of engineered circuits, and their ecological effects during prolonged residence in the human microbiome, remain insufficiently characterized.

The second factor is regulatory classification, which depends strongly on intended use. Microorganisms marketed as dietary supplements follow substantially different development pathways from products intended to prevent or treat disease. The latter are generally developed as live biotherapeutic products (LBPs) and must meet pharmaceutical standards for manufacturing, consistency, safety and clinical efficacy (Duhan et al., 2025; Zhang et al., 2026). Organisms with similar biological properties may therefore face very different pathways depending on their claims and regulatory context, with engineered strains requiring the most extensive characterization. These requirements extend beyond the organism to chemistry, manufacturing and controls: strain identity and genetic stability across production, reproducible potency assays, consistent viable counts, and release and stability testing appropriate to a living product (US Food and Drug Administration, 2016). Regulatory requirements and classification frameworks differ across jurisdictions, including between the United States and Europe, but engineered strains have progressed to Phase 3 under existing regulatory frameworks, demonstrating that development pathways exist in these jurisdictions (Das, 2026). What does not yet exist in any jurisdiction is an approved engineered live biotherapeutic. Thus, the limitation reflected in our framework is one of regulatory precedent, rather than the absence of a regulatory pathway.

### 5.2 Ecological complexity and engraftment

A pattern emerges across both niches examined here: more advanced products increasingly use donor-derived communities or multiple strains rather than a single organism. Rebyota and Vowst are donor-derived and spore-based consortia respectively, and both are approved for rCDI, whereas no single-strain product has been approved for this indication (Feuerstadt et al., 2022; Khanna et al., 2022). In the vaginal niche, multi-strain approaches have also shown evidence of establishment, although the reported endpoints differ across studies and are not directly comparable (Ravel et al., 2025; Potloane et al., 2026). Further consortia are in clinical development for indications including graft-versus-host disease and inflammatory bowel disease (Sorbara and Pamer, 2022).

Several mechanisms could account for this. Consortia may provide functional redundancy and cross-feeding, occupy multiple ecological niches and increase the likelihood that at least one member is compatible with the recipient’s resident community. The latter is supported by evidence that LACTIN-V colonization depended on the pretreatment microbiome and inflammatory state (Bloom et al., 2026). Ecological complexity may therefore help address the colonization constraint identified by our framework.

### 5.3 The readiness framework

**Figure 1** presents a translational readiness framework, showing that successful clinical translation depends on whether a product can satisfy a set of interconnected requirements.

**Figure 1:**
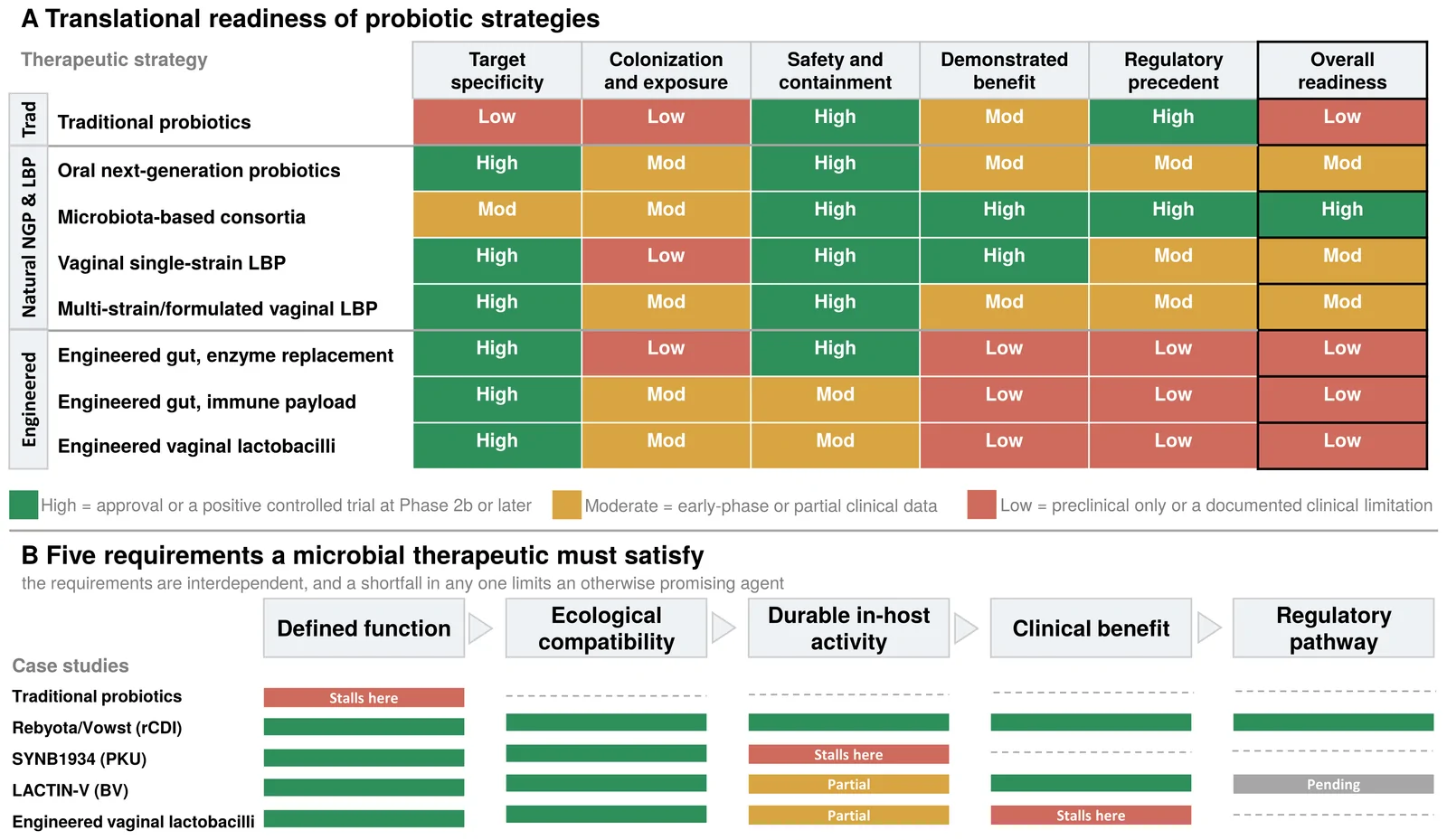
Translational-readiness framework for probiotic therapeutics. **(A)** Qualitative comparison of traditional probiotics, natural next-generation probiotics (NGPs), live biotherapeutic products (LBPs), microbiota-based consortia and engineered microbial therapeutics across five factors: target specificity, colonization and exposure, safety and containment, demonstrated benefit and regulatory precedent, together with an overall readiness estimate. High indicates regulatory approval or a positive controlled trial at Phase 2b or later, moderate indicates early-phase or partial clinical evidence and low indicates preclinical evidence or a documented clinical limitation. Ratings are aggregate across each strategy’s principal indications and may differ for individual indications. Demonstrated benefit grades the strength of positive, indication-specific evidence rather than the volume of research activity. Containment applies only to engineered strains; for other products the rating reflects an established safety record and the absence of a containment requirement. Regulatory precedent reflects whether products in the class have been approved. Overall readiness is anchored on demonstrated benefit and adjusted downward where other dimensions impose material limitations; it is not an average, so a strategy may rate high on safety and regulatory precedent yet remain low overall if specificity or colonization is limiting. **(B)** Five interdependent requirements for translation: a defined function, ecological compatibility, durable in-host activity, clinical benefit and a regulatory pathway, illustrated using representative case studies. Rebyota/Vowst satisfy all five requirements, whereas traditional probiotics, SYNB1934, LACTIN-V and engineered vaginal lactobacilli remain limited at different stages of development. rCDI, recurrent *Clostridioides difficile* infection; PKU, phenylketonuria; BV, bacterial vaginosis.

Panel A compares therapeutic strategies across five dimensions: target specificity, colonization and exposure, safety and containment, demonstrated benefit and regulatory precedent. It shows that greater biological sophistication does not necessarily mean greater clinical maturity and that different products may be limited by different factors. Overall readiness is anchored on demonstrated benefit and adjusted downward where other dimensions impose material limitations; it is not an average, so a strategy may rate high on safety and regulatory precedent yet remain low overall if specificity or colonization is limiting.

Ratings follow a stated rule: high denotes regulatory approval or a positive controlled trial at Phase 2b or later, moderate denotes early-phase or partial clinical data, and low denotes preclinical-only status or a documented clinical limitation. Demonstrated benefit grades the strength of positive, indication-specific evidence rather than the volume of research activity, and ratings are aggregate across each strategy’s principal indications, so individual indications may score differently. Containment applies only to engineered strains; for other products the rating reflects an established safety record and the absence of a containment requirement. Regulatory precedent reflects whether products in the class have been approved. The framework is qualitative, derived from cases reviewed here, and has not been externally validated; it is intended to structure prioritization rather than to predict outcomes. Panel B illustrates the five requirements underlying this comparison. First, the therapeutic function must be clearly defined and linked to a clinically relevant target. Second, the therapeutic must function within a compatible ecological and host environment. This can depend on baseline microbiome composition, inflammation, prior treatment and competing organisms. Third, the product must reach the intended site and remain active long enough to produce benefit. This may require durable colonization, although transient activity, repeated administration or broader restructuring of the microbial community may be sufficient in some cases. Fourth, biological activity must translate into meaningful clinical benefit; target engagement, microbial detection and changes in community composition are important intermediate outcomes, but do not demonstrate clinical efficacy on their own. Finally, the product must have a feasible development pathway, including safety, biocontainment where relevant, genetic and phenotypic stability, reproducible manufacturing, formulation, potency testing and regulatory classification.

## 6 Conclusion

Natural and engineered probiotics are becoming increasingly sophisticated, but their clinical maturity is better judged by progress toward implementation than by mechanistic novelty. Microbiota-based therapies have reached approval for rCDI, where a defined pathogen, treatment window, delivery strategy and clinical endpoint aligned. Engineered strains have not yet reached this stage. SYNB1934 demonstrated human target engagement without clinical benefit, while studies of *A. muciniphila* showed that treatment response may depend on the recipient’s baseline microbiome.

These examples show that microbial function alone is insufficient. A therapeutic must remain active at the intended site for the required duration, operate within a compatible ecological context and produce a meaningful clinical benefit. Durable colonization is essential for some restorative approaches, though transient activity or repeated dosing may suffice for others. The vaginal niche illustrates how strain selection, multi-strain products, formulation and release kinetics can be used to improve microbial establishment.

The framework supports prioritization by highlighting the factors most likely to determine translational readiness. Across applications, target specificity, colonization and exposure, safety and containment, demonstrated benefit and regulatory precedent emerge as recurring barriers to translation. Future trials should account for baseline features that predict microbial establishment, define the duration of in-host activity required for each therapeutic strategy and integrate safety and regulatory planning early in development.
